# Enhancement of Recombinant Protein Production in Transgenic *Nicotiana benthamiana* Plant Cell Suspension Cultures with Co-Cultivation of *Agrobacterium* Containing Silencing Suppressors

**DOI:** 10.3390/ijms19061561

**Published:** 2018-05-24

**Authors:** Ting-Kuo Huang, Bryce W. Falk, Abhaya M. Dandekar, Karen A. McDonald

**Affiliations:** 1 Department of Chemical Engineering and Materials Science, University of California, 1 Shields Avenue, Davis, CA 95616, USA; huang.tingkuo@gene.com; 2 Department of Plant Pathology, University of California, 1 Shields Avenue, Davis, CA 95616, USA; bwfalk@ucdavis.edu; 3 Department of Plant Sciences, University of California, 1 Shields Avenue, Davis, CA 95616, USA; amdandekar@ucdavis.edu

**Keywords:** α1-antitrypsin, inducible promoter, viral amplicon, gene silencing suppressor, post-transcriptional gene silencing, transgenic plant cell cultures

## Abstract

We have previously demonstrated that the inducible plant viral vector (CMViva) in transgenic plant cell cultures can significantly improve the productivity of extracellular functional recombinant human alpha-1-antiryspin (rAAT) compared with either a common plant constitutive promoter (*Cauliflower mosaic virus* (CaMV) 35S) or a chemically inducible promoter (estrogen receptor-based XVE) system. For a transgenic plant host system, however, viral or transgene-induced post-transcriptional gene silencing (PTGS) has been identified as a host response mechanism that may dramatically reduce the expression of a foreign gene. Previous studies have suggested that viral gene silencing suppressors encoded by a virus can block or interfere with the pathways of transgene-induced PTGS in plant cells. In this study, the capability of nine different viral gene silencing suppressors were evaluated for improving the production of rAAT protein in transgenic plant cell cultures (CMViva, XVE or 35S system) using an *Agrobacterium*-mediated transient expression co-cultivation process in which transgenic plant cells and recombinant *Agrobacterium* carrying the viral gene silencing suppressor were grown together in suspension cultures. Through the co-cultivation process, the impacts of gene silencing suppressors on the rAAT production were elucidated, and promising gene silencing suppressors were identified. Furthermore, the combinations of gene silencing suppressors were optimized using design of experiments methodology. The results have shown that in transgenic CMViva cell cultures, the functional rAAT as a percentage of total soluble protein is increased 5.7 fold with the expression of P19, and 17.2 fold with the co-expression of CP, P19 and P24.

## 1. Introduction 

Post-transcriptional gene silencing (PTGS) is a defense mechanism of plants against invading foreign nucleic acids such as viral infection and transgene expression in plant cells [1,2,3]. In this PTGS process, double-stranded short-interfering RNA (ds siRNA, 21–25 nt) is cleaved from double-stranded RNA (dsRNA) [4] or single-stranded RNA (ssRNA) viral sequences or transgenes by RNase III-type enzyme DICER [5]. The generation of these dsRNA is thought to originate during viral replication, and/or from internal pairing of long RNA assisted by RNA-dependent RNA polymerase (RdRP) [6]. These ds siRNA molecules are unwound by RNA helicase to form single-stranded short-interfering RNA (ss siRNA), and then assembled into the host RNA-induced silencing complex (RISC) to form a siRNA-RISC complex. The siRNA-RISC complex utilizes siRNA as guides for targeting and cleaving sequence-complementary RNA molecules (complementary to the siRNA sequences which are parts of the viral or transgene transcripts) [6,7,8,9]. Therefore, the sequence-complementary mRNA molecules are degraded by the siRNA-RISC complex machinery, and the transgene expression is silenced post-transcriptionally (i.e., mRNA cannot be translated into protein).

Plant viruses encode specific proteins, known as viral gene silencing suppressors, which can block or interfere with the plant host RNA silencing processes based on different modes of action at various steps in the pathways of transgene-induced post-transcriptional gene silencing in plant cells [1,3,10]. More than twenty post-transcriptional gene silencing suppressors have been discovered [1] since HC-Pro, with the first gene silencing suppressor being identified in 1998 [11]. Although the exact mechanism of these suppressors is unknown, their functions have been recently investigated [3,12]. For example, the HC-Pro suppressor of the *Tobacco etch virus* (TEV) acts by inhibiting the unwinding step of ds siRNA molecules and the RISC assembly [13]. The P19 suppressor from *Tomato bushy stunt virus* (TBSV) and the P21 protein of *Beet yellows virus* (BYV) target and interact with ds siRNA molecules directly, preventing them from being processed or incorporated into the siRNA-RISC machinery [3,14]. 

*Agrobacterium*-mediated transient expression is a rapid and flexible approach to produce recombinant proteins of interest [15,16,17,18]. Recombinant *Agrobacterium tumefaciens* strains can be used for transient expression of transgenes that have been inserted into the T-DNA region of the Ti plasmid in *Agrobacterium* [19]. *Agrobacterium*, which infiltrates plant tissues, mediates transgene transfer from the T-DNA region of the Ti-plasmid into the plant cells [19,20]. In this case, the transgene of interest is not integrated into the genome of plant cells. The plant cells infected by recombinant *Agrobacterium* can transiently express the transgene for a couple of days (4–14 days, depending on the type of recombinant protein, host and expression system). A further advantage of the *Agrobacterium* infiltration system is its capability to transfer several transgenes into the same plant host cell, so that multimeric proteins, such as antibodies, can be expressed and assembled [21]. Investigations have demonstrated that the co-expression of viral gene silencing suppressors can significantly prevent the onset of transgene-induced PTGS, and enhance high expression level of transgene in plant leaves through an *Agrobacterium*-mediated transient expression process [22,23,24,25].

In previous studies, it has been shown that the functional rAAT productivity in transgenic plant cell cultures can be significantly improved by using the CMViva system compared with either a plant constitutive promoter (35S) or a chemically inducible promoter (XVE) system [26,27]. In this study, the effectiveness of nine different viral gene silencing suppressors (Table 1), which have different modes of action, were investigated for their ability to interfere with transgene-induced PTGS in order to enhance the expression of the recombinant human α1-antitrypsin (rAAT) in transgenic *Nicotiana benthamiana* cell cultures. The viral gene silencing suppressors were introduced into the plant cell host using an *Agrobacterium*-mediated transient expression co-cultivation process, in which the stably transgenic plant cell cultures expressing rAAT protein driven by CMViva, XVE or 35S system were co-cultured with recombinant *Agrobacteria* carrying the viral gene silencing suppressor. The chemically inducible estradiol-activated XVE system has been developed for regulating transgene expression, which is activated by using estradiol as inducer, in transgenic plants [28]. We have developed a novel CMV inducible viral amplicon (CMViva) expression system; it has been demonstrated that the CMViva system allows tightly regulated expression of the transgene and functional human protein production in transgenic plant cell culture [26,27], and in plant hosts by utilizing transient agroinfiltration [25]. The CMViva system encodes a viral replicase, which is tightly controlled by the XVE promoter system, along with other engineered modifications, so that the recombinant viral amplicons of the CMViva system are only expressed intracellularly under induction conditions.

The impact of viral gene silencing suppressors on rAAT expression within transgenic cell cultures was characterized according to an improvement in extracellular rAAT production yield and functionality. To develop the *Agrobacterium*-mediated transient expression co-culture process, the effects of co-culture conditions for optimal gene transfer efficiency on rAAT production were investigated first. The purpose of this study is to investigate the onset of PTGS in transgenic cell culture (CMViva, XVE and 35S) expressing rAAT protein, and to identify the potential of using viral gene silencing suppressors to improve rAAT expression. The synergetic effect of multiple viral gene silencing suppressors was further investigated using design of experiment (DOE) methodology. 

## 2. Results 

### 2.1. Development of Transient Expression Co-Cultivation Process

To develop a reliable and reproducible transient expression co-culture process, critical co-culture conditions were investigated in this study, including (1) temperature effect; (2) biomass ratio of *Agrobacterium* to plant cells; (3) effect of timing of starting the co-cultivation process (related to the physiological status of plant cells to be agroinfiltrated); and (4) effect of induction timing after starting the co-cultivation process. Recombinant *Agrobacterium* carrying the viral gene silencing suppressor P19 and the transgenic CMViva cell culture were chosen as a model system to evaluate these co-culture conditions, and to develop the co-cultivation process. These initial screening experiments were performed with only one replicate to identify appropriate starting conditions; further experiments using these starting conditions were replicated.

**Figure 1:**
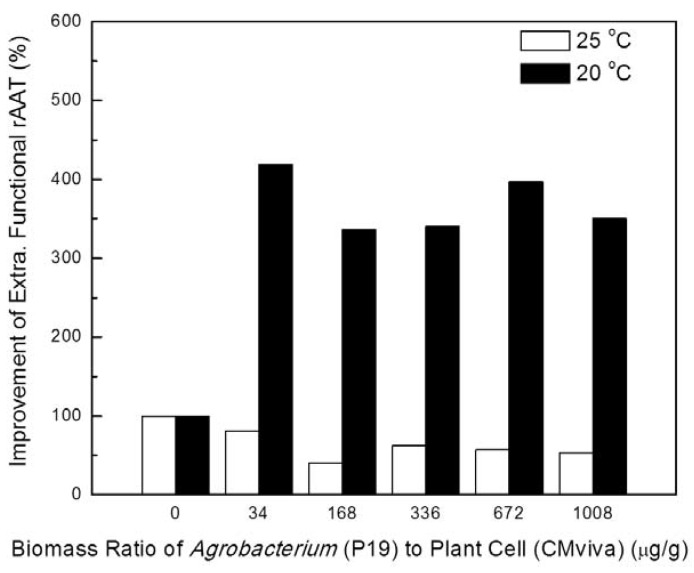
The effect of biomass ratio (0, 34, 168, 336, 672 and 1008 μg-DCW bacterial cell/g-DCW plant cell) of *Agrobacterium* (P19) to suspended transgenic plant cells (CMViva) co-cultivated in a 6 well-microplate on the improvement of functional rAAT protein production at 20 °C or 25 °C. DCW stands for dry cell weight. Transgenic CMViva cell culture without the co-cultivation of recombinant *Agrobacterium* was used as the control. Samples were taken on day 6 post-induction.

**Figure 2:**
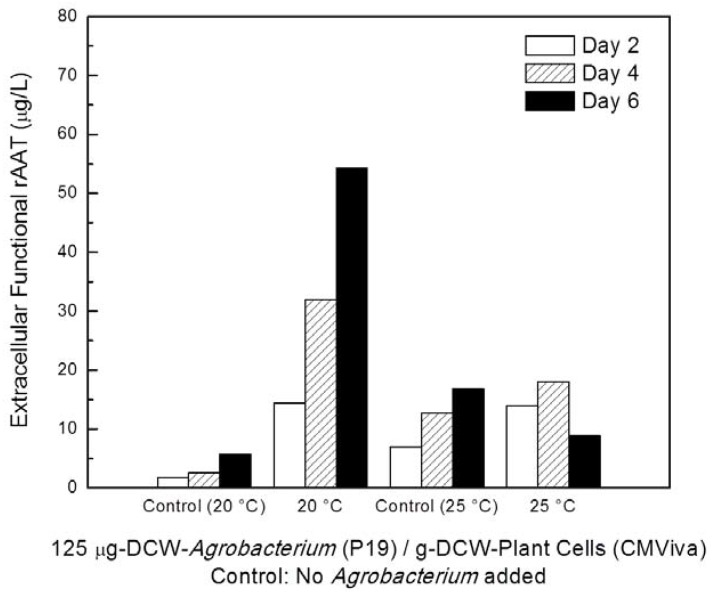
The kinetics of functional rAAT protein production for transgenic plant cells (CMViva) co-cultivated with *Agrobacterium* (P19) in a 6 well-microplate at 20 °C or 25 °C. Transgenic CMViva cell culture without the co-cultivation of recombinant *Agrobacterium* was investigated as control. The biomass ratio of *Agrobacterium* to plant cells was 125 μg-DCW/g-DCW. Samples were taken on days 2, 4 and 6 post-induction.

Temperature dramatically affects plant-virus interactions, leading to interferences with virus-induced or transgene-induced PTGS [37,38,39]. Two temperature conditions during the co-culture process were tested: 25 °C and 20 °C. Recombinant *Agrobacterium* carrying viral gene silencing suppressor P19 and transgenic CMViva plant cells were co-cultured in a 6-well microplate in the dark at different biomass ratios (based on dried cell weight) of *Agrobacterium* to plant cell. The inducer was added to initiate the rAAT gene expression on day 1 after co-cultivation. Samples were taken on days 2, 4 and 6 post-induction. Figure 1 and Figure 2 show the effect of temperature on the transfection efficiency of *Agrobacterium* (P19) cells into suspended plant cell cultures (CMViva). At a lower temperature (20 °C), higher extracellular functional rAAT was achieved when compared with co-cultivation at room temperature (25 °C), even though without the *Agrobacterium* (P19) addition, higher extracellular functional rAAT was obtained when the transgenic cells were incubated at the higher temperature (25 °C).

**Figure 3:**
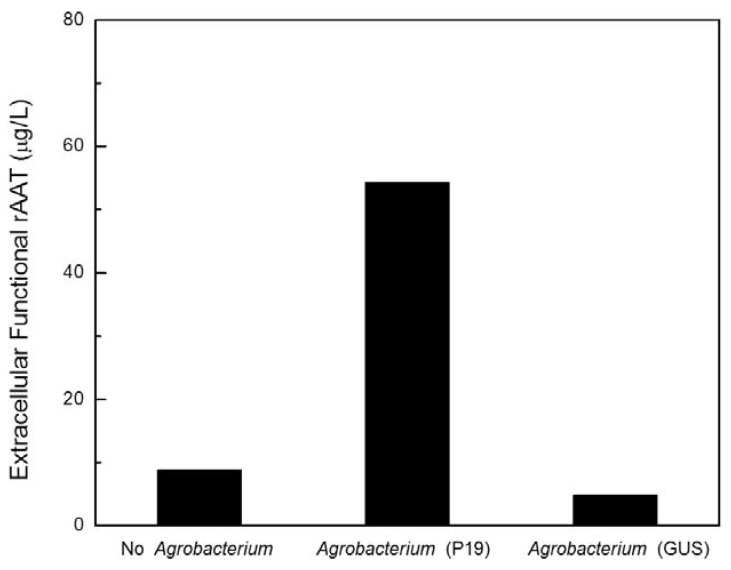
Recombinant human AAT production in transgenic CMViva cell cultures co-cultivated with either recombinant *Agrobacterium* strain (containing 35S CaMV promoter driving GUS expression) or recombinant *Agrobacterium* strain carrying the P19 viral gene silencing suppressor in a 6-well microplate at 20 °C. The biomass ratio of *Agrobacterium* to plant cells is 125 μg-DCW/g-DCW. Samples were taken 6 days post-induction.

To confirm that the enhanced production of functional rAAT in transgenic CMViva culture was attributed to the transient expression of viral gene silencing suppressor P19 during the co-culture process, the recombinant *Agrobacterium* EHA105 strain (containing 35S CaMV promoter driving GUS (β-glucuronidase) expression) and recombinant *Agrobacterium* EHA105 strain carrying the viral gene silencing suppressor P19 were separately co-cultured with transgenic CMViva cell culture for comparison. Transgenic CMViva cell culture without the co-cultivation of any *Agrobacterium* solution was also investigated as a control, in a 6-well microplate at 20 °C. The inducer was added on day 1 after co-cultivation, and samples were taken on day 6 post-induction. Figure 3 shows that the improvement of functional rAAT was only observed for co-culture with *Agrobacterium* containing the gene for the silencing suppressor P19, indicating that the improvement was likely due to the transient expression of P19 in the plant cells during the co-culture process, and not to interactions with the *Agrobacterium* cultures or the plant cells’ response to the co-cultivation process.

**Figure 4:**
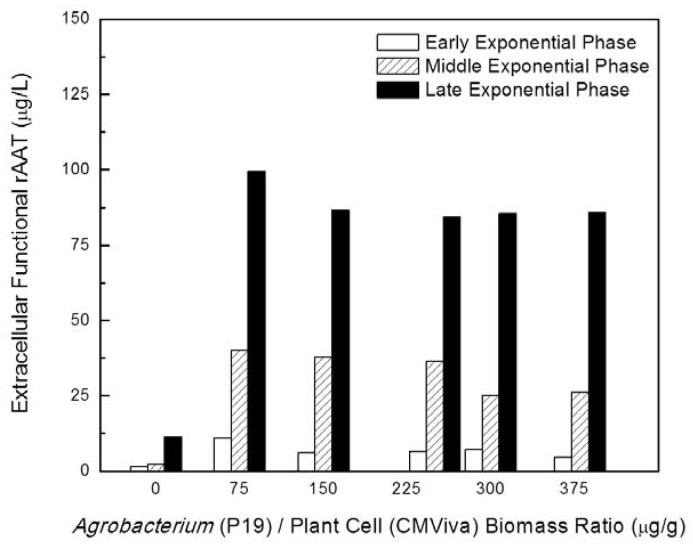
Effect of timing of starting co-cultivation process (physiological status of plant cells to be agroinfiltrated) on enhancing rAAT production. Recombinant *Agrobacterium* carrying viral gene silencing suppressor P19 was added to transgenic CMViva cell culture at different physiological states of plant cells, including the early-exponential, mid-exponential and late-exponential growth phases, under different biomass ratios (0, 75, 150, 250, 300 and 380 µg/g) of *Agrobacterium* to plant cells in shake flask at 20 °C. Samples were taken on day 6 post-induction.

**Figure 5:**
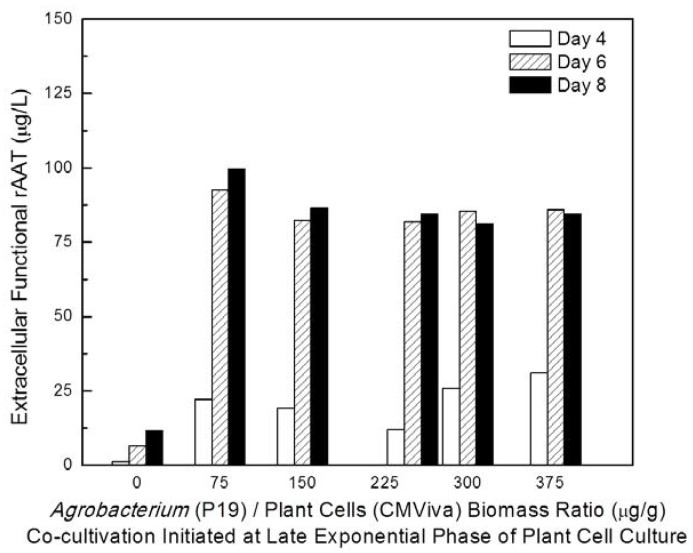
Time course of functional rAAT production in transgenic CMViva cells co-cultivated with recombinant *Agrobacterium* carrying P19. Co-cultivation was initiated in the late exponential phase of plant cell culture, with a variety of biomass ratio (0, 75, 150, 250, 300 and 380 µg/g) of *Agrobacterium* to plant cells in shake flasks at 20 °C. Samples were taken on days 4, 6 and 8 post-induction.

**Figure 6:**
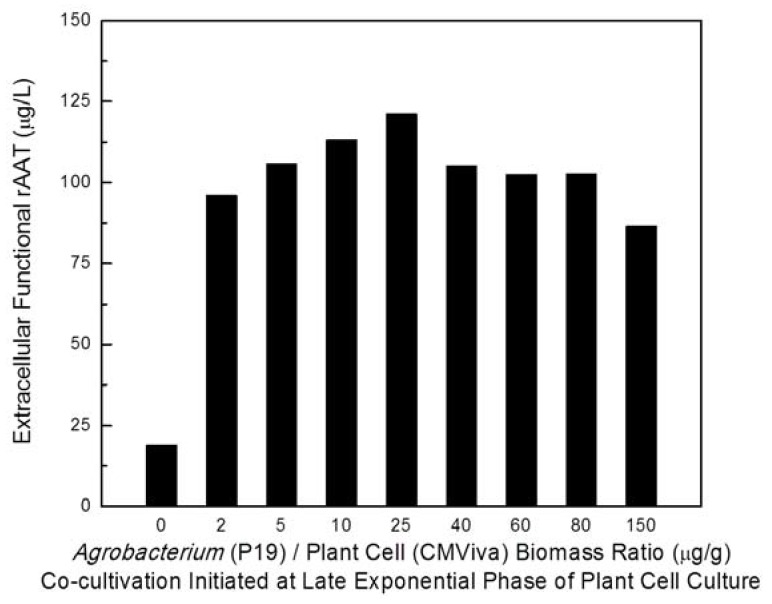
Effect of biomass ratio (0, 2, 5, 10, 25, 40, 60, 80 and 150 µg/g) of *Agrobacterium* to transgenic plant cells (CMViva) on rAAT production in shake flasks at 20 °C. Co-cultivation was initiated in the late exponential phase of plant cell culture. Samples were taken on day 8 post-induction.

To understand the effect of variations in the timing of starting the co-cultivation process (physiological status of plant cells to be agroinfiltrated) on enhancing rAAT production, recombinant *Agrobacterium* culture carrying the viral gene silencing suppressor P19 was co-cultured with transgenic CMViva cell culture in shake flasks at 20 °C at different plant cell growth states, including early-exponential, mid-exponential, and late-exponential growth phases, with different *Agrobacterium* to plant cell biomass ratios. The inducer was added one day after co-cultivation. Figure 4 (samples were taken on day 6 post-induction) clearly indicates that *Agrobacterium* co-cultivation initiated at the late-exponential phase of the plant cell culture resulted in a higher functional rAAT production yield. Also, these data indicated that an *Agrobacterium* to plant biomass ratio of 75 µg/g is sufficient for a maximally beneficial effect. Figure 5 shows the rAAT production kinetics at various time points post-induction (days 4, 6 and 8) for co-cultivation initiated at the late exponential phase for various *Agrobacterium* to plant biomass ratios. These data confirm thon days 6-8 post induction, at a ratio of 75 µg/g, provide the highest extracellular functional rAAT levels. Furthermore, a detailed investigation regarding the effect of the biomass ratio of *Agrobacterium* to plant cells on rAAT production at 20 °C in shake flasks is shown in Figure 6 (samples were taken on day 8 post-induction), indicating that the optimal biomass ratio of *Agrobacterium* to plant cell is in the range of 5 to 80 μg-DCW/g-DCW. 

**Figure 7:**
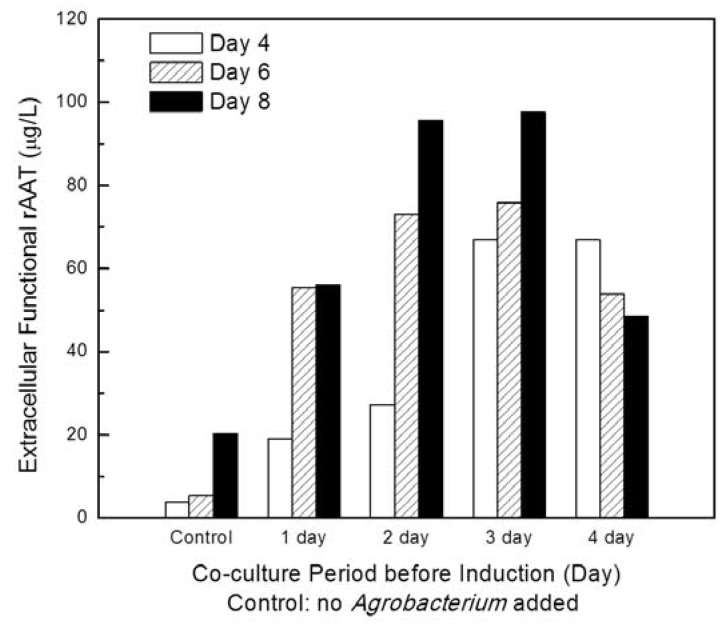
Investigations of the effect of induction timing after starting co-cultivation process on rAAT production. The inducer was added into the co-culture process of *Agrobacterium* carrying viral gene silencing suppressor P19 with transgenic CMViva on days 1, 2, 3 and 4 after agroinfiltration in a 6-well microplate at 20 °C. The biomass ratio of *Agrobacterium* to plant cell was 25 μg-DCW/g-DCW. Samples were taken on days 4, 6 and 8 post-induction.

To further understand the impact of induction timing after starting the co-cultivation process, and the possibility of onset of PTGS in transgenic plant cell culture before induction, the inducer was added into the co-culture process of *Agrobacterium* carrying viral gene silencing suppressor P19 with transgenic CMViva on days 1, 2, 3 and 4 after co-cultivation, in a 6-well microplate at 20 °C. The biomass ratio of *Agrobacterium* to plant cell was 25 μg-DCW/g-DCW. Samples were taken on days 4, 6 and 8 post-induction. Figure 7 shows that an induction phase started on day 2 or 3 after the addition of the *Agrobacterium* carrying the P19 gene led to a higher extracellular functional rAAT production yield.

Through the evaluation of critical co-culture conditions for enhancing functional rAAT production, the optimal conditions for developing transient expression co-culture process include (1) co-cultivation and recombinant protein production at 20 °C; (2) biomass ratio of *Agrobacterium* to plant cells at 5 to 80 μg-DCW/g-DCW; (3) starting the co-cultivation using plant cells in the late-exponential growth phase; and (4) adding the chemical inducer on day 2 after the bacterial cells and plant cells are mixed. 

### 2.2. Effect of Viral Gene Silencing Suppressors on rAAT Production in Transgenic 35S, XVE and CMViva Cell Cultures 

To determine optimal transient expression co-culture conditions, nine different viral gene silencing suppressors (Table 1) were chosen to evaluate their potential to enhance extracellular rAAT production in transgenic 35S cell culture, XVE cell culture, and CMViva cell culture, through the inhibition of PTGS using the transient expression co-culture approach. Recombinant *Agrobacterium* strains transformed with viral gene silencing suppressors were individually co-cultivated with transgenic 35S, XVE, and CMViva cell culture in shake flasks at 20 °C with a 25 μg-DCW/g-DCW biomass ratio of *Agrobacterium* to plant cells. Samples were taken on day 8 post-induction. The transgenic 35S, XVE and CMViva cell culture alone (without co-cultivation of agrobacteria) was also tested as a control for comparison. 

**Figure 8:**
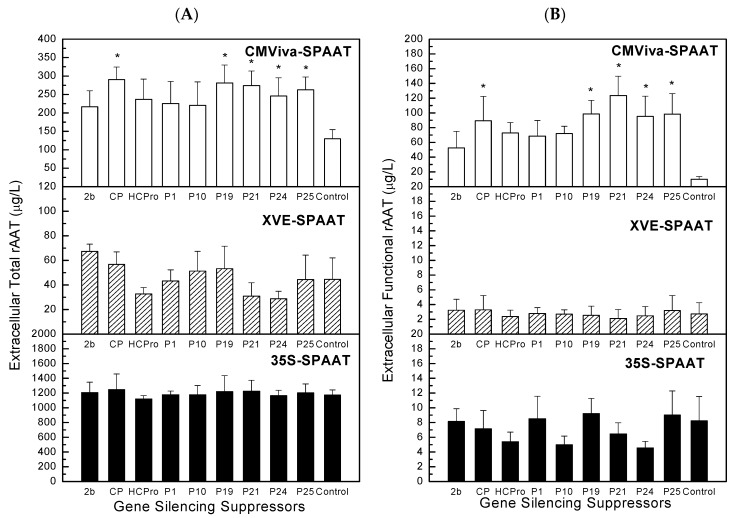

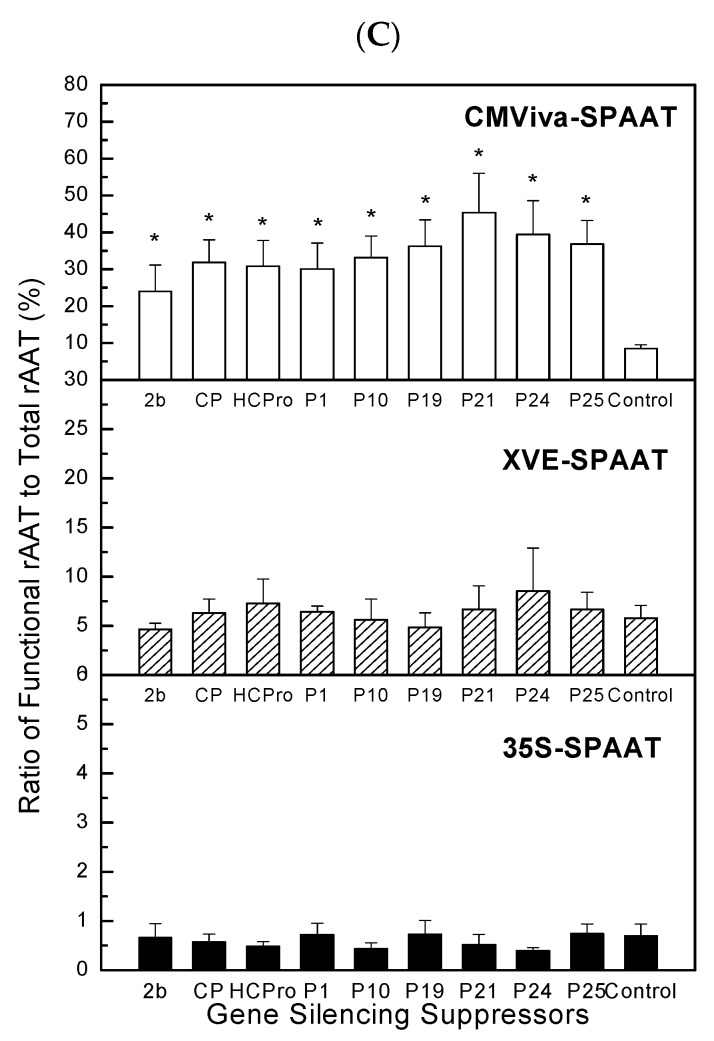
Effect of viral gene silencing suppressors on extracellular total rAAT production (**A**), extracellular functional rAAT production (**B**), and ratio of functional rAAT to total rAAT (**C**) in transgenic plant cell cultures (CMViva, XVE or 35S). Error bars represent one standard deviation of measurements obtained from triplicate sample assays of duplicate experiments. Asterisks on the graphs indicate a statistically significant difference from the control (*p* ≤ 0.05).

Interestingly, Figure 8 (total rAAT yield, functional rAAT yield, and ratio of functional rAAT to total rAAT) shows that CP, P19, P21, P24, and P25 were viral gene silencing suppressors that significantly enhanced the production of the extracellular total rAAT and functional rAAT (statistically significant by Student’s *t* test with *p* ≤ 0.05). For 35S and XVE cell culture, Figure 8 indicates that statistically insignificant differences were observed between results obtained using viral gene silencing suppressors and the control, suggesting that viral gene silencing suppressors do not improve the production of extracellular total and functional rAAT in transgenic 35S cell cultures and XVE cell cultures (improvements were shown to be statistically insignificant by a Student’s *t* test with *p* > 0.05).

### 2.3. Impact of Co-Expression of Viral Gene Silencing Suppressors on rAAT Production Using Design of Experiment

In this study, viral gene silencing suppressor candidates were chosen to investigate the possible synergistic effects of multiple viral gene silencing suppressors on rAAT production in the transgenic CMViva cell culture, and on inhibiting transgene-induced PTGS through different modes of action. The five identified viral gene silencing suppressors CP, P19, P21, P24 and P25, all of which were shown to have positive effects on rAAT production in transgenic CMViva cell cultures (Figure 8), were selected for these studies. The design of experiments, including fractional factorial design (for screening significant variables) and response surface methodology using central composite design (for optimizing the values of significant variables), were applied to efficiently evaluate the synergistic effects of viral gene silencing suppressors. 

Fractional factorial experimental design was first applied to screen and identify which variables (i.e., viral gene silencing suppressors) were able to enhance rAAT production. Five variables (CP, P19, P21, P24 and P25), with two levels (high and low biomass ratio of *Agrobacterium* to plant cells), were screened, and their effects were evaluated according to the experimental design matrix shown in Table 2. Recombinant *Agrobacterium* strains transformed with the viral gene silencing suppressor of interest were co-cultivated with eight-day-old transgenic CMViva cell cultures in a shake flask at 20 °C, with a 10 μg-DCW/g-DCW biomass ratio of *Agrobacterium* to plant cell. After two days of co-cultivation, an estradiol inducer was added into the plant cell cultures to initiate the rAAT expression. Samples were taken on day 6 post-induction. The transgenic CMViva cell culture alone (without co-cultivation with agrobacteria) was also tested as a control for comparison. Table 2 represents the fractional factorial design, with two levels of each variable and the corresponding rAAT production as responses. Apparently, the transient expression obtained by combining specific viral gene silencing suppressors can further enhance the rAAT production yield and functionality (Table 2). 

Next, three viral gene silencing suppressor candidates (CP, P19, and P24, Run 12 in Table 2, since those conditions gave the highest extracellular functional rAAT) were then chosen (confidence levels were accepted only when above 95%, *p* ≤ 0.05) for further optimization of their values (biomass ratio of *Agrobacterium* to plant cells) to maximize rAAT yield and functionality, using a central composite design (CCD) combined with one central point and one control point, for a total 16 runs (Table 3). Each variable was investigated at five coded levels (+2, +1, 0, −1, −2). Recombinant *Agrobacterium* strains transformed with viral gene silencing suppressor of interest were co-cultivated with eight-day-old transgenic CMViva cell cultures in a shake flask at 20 °C, with a specific μg-DCW/g-DCW biomass ratio of *Agrobacterium* to plant cell (Table 3). After two days of co-cultivation, an estradiol inducer was added into the plant cell cultures to initiate the rAAT expression. Samples were taken on day 6 post-induction. The transgenic CMViva cell culture alone (without co-cultivation with agrobacteria) was also tested as a control for comparison.

**Figure 9:**
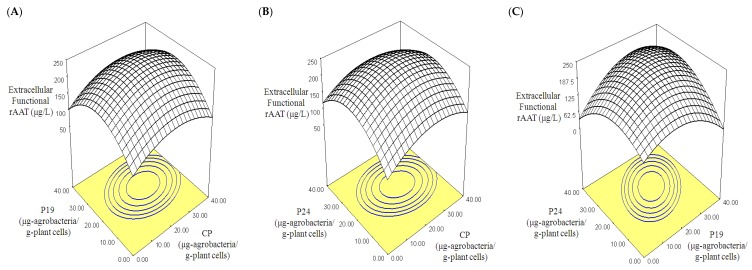
Response surfaces and contour plots (**A**–**C**) for response of extracellular functional rAAT production in transgenic CMViva cell culture (samples were taken on day 6 post-induction) with a variety of biomass ratio of *Agrobacterium* carrying viral gene silencing suppressors (CP, P19, or P24) to plant cells (μg-*Agrobacterium*/g-Plant cells), showing the strong interactive effects.

Table 3 shows the CCD matrix of three variables, along with experimental and predicted values of rAAT production yield as responses. Figure 9 presents the response surfaces for the response of extracellular functional rAAT production to the different biomass ratio of *Agrobacterium* carrying the viral gene silencing suppressor to plant cells (μg-DCW/g-DCW); this shows that there are well-defined optimal concentrations for candidates of recombinant *Agrobacterium* carrying specific viral gene silencing suppressors, and also illustrates the existence of interactions. The experimental optimum biomass ratio (*Agrobacterium* to plant cells) of CP, P19, and P24 for maximizing the rAAT production yield (618.4 μg-(extracellular total rAAT)/L) and functionality (301.6 μg-(extracellular functional rAAT)/L) was 25 μg-DCW/g-DCW in all three viral gene silencing suppressors (i.e., 1:1:1, Run 8 in Table 3).

**Figure 10:**
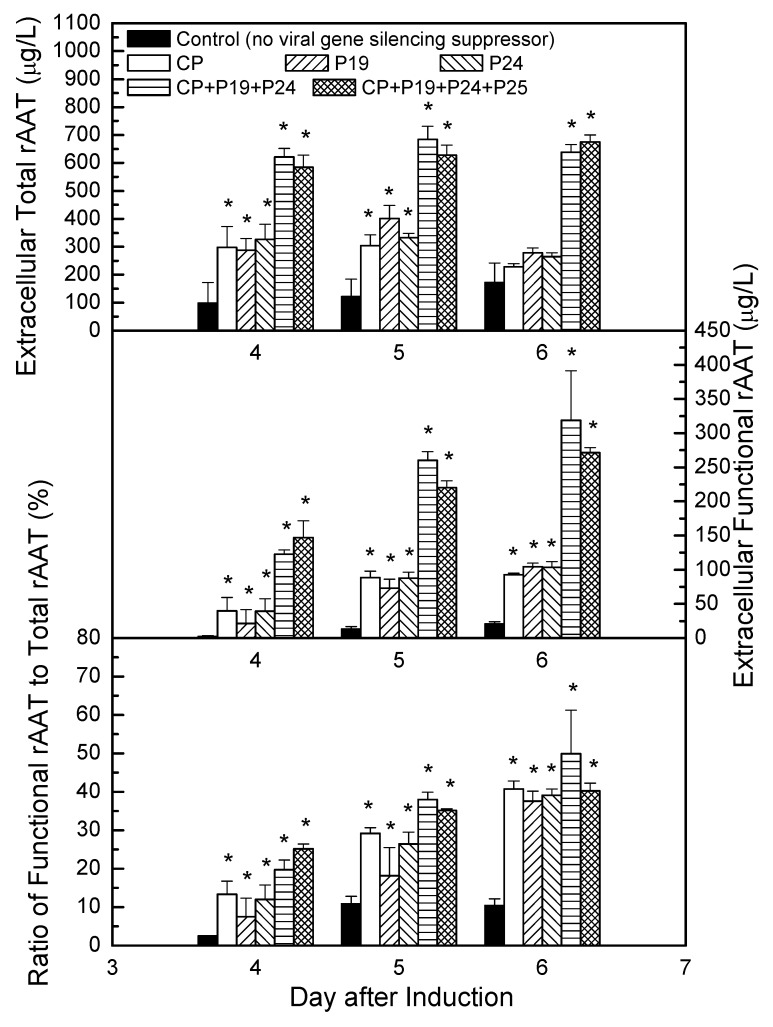
Comparisons of the rAAT yield and functionality in transgenic CMViva cell culture co-cultivated with individual or combined recombinant *Agrobacterium* carrying viral gene silencing suppressor (CP, P19, P24 or P25). Error bars represent one standard deviation of measurements obtained from triplicate sample assays of duplicate experiments. An asterisk shown on the graphs indicates that there is a statistically significant difference from the control group (*p* ≤ 0.05).

The time course of the improvements on rAAT production yield and functionality in transgenic CMViva cell cultures by co-cultivating with recombinant *Agrobacterium* are presented in Figure 10. Each recombinant *Agrobacterium* carrying viral gene silencing suppressor (CP, P19, P24 or P25) was co-cultivated with transgenic CMViva cell culture at a biomass ratio of 25 μg/g in shake flasks at 20 °C. After two days of co-cultivation, an estradiol inducer was added into the plant cell cultures to initiate the rAAT expression. Samples were taken on days 4, 5, and 6 post-induction. Apparently, there are positive effects on improving rAAT production yield and functionality in experiments combining CP, P19, and P24, compared with individual CP, P19, or P24 and the control experiment.

## 3. Discussion

In this study, we developed an *Agrobacterium*-mediated transient expression co-cultivation process by growing plant cells and a recombinant *Agrobacterium* strain in a suspension environment, in order to evaluate the capability of transient production of viral gene silencing suppressors (Table 1) for improving the rAAT production in transgenic plant cell cultures (CMViva, XVE or 35S system). Through the co-culture process, the impacts of viral gene silencing suppressors on the production of recombinant protein in transgenic plant cell cultures were determined, and useful viral gene silencing suppressors were then identified. In addition, the synergistic effects of different viral gene silencing suppressors were further optimized using the statistical design of experiment. 

Recombinant AAT production was significantly increased at a lower co-cultivation temperature (20 °C) compared to room temperature (25 °C) (Figure 1 and Figure 2), suggesting that lower temperatures may inhibit *Agrobacterium* bacterial growth, leading to a delay in the death of plant cells, or it may enhance T-DNA transfer from the *Agrobacterium* to the plant cells. We next confirmed that the improvement in rAAT production is due to the viral gene silencing suppressor expression, instead of the presence of *Agrobacterium* or the plant cells’ response to the infiltration process (Figure 3), indicating that the viral gene silencing suppressor gene can be transiently delivered to plant cells and then successfully transcribed and translated, and can execute its function to suppress the pathway of post-transcriptional gene silencing. Furthermore, the impact of the ratio of recombinant *Agrobacterium* to transgenic plant cells on rAAT production was evaluated. Higher concentrations of *Agrobacterium* (>1000 μg/g) showed lower rAAT production; this is likely due to the introduction of saturating levels of bacteria leading to rapid senescence (and browning) of the plant cell cultures. 

Recently studies have demonstrated that large-scale *Agrobacterium*-mediated transient expression systems can be performed using vacuum agroinfiltration of whole plant tissues. The closed, contained system, and controllable process conditions in a plant cell culture bioreactor process allow optimization of consistent, reproducible production of human therapeutics. Because the plant cells are in the form of small aggregates in a liquid suspension, there is a high level of accessibility of the *Agrobacterium* to the plant cell surface. Furthermore, the physiological states of both the *Agrobacterium* and the plant cell can be independently optimized, as well as the co-cultivation conditions, which may provide better reproducibility and high-level heterologous protein expression in plant cell culture [40]. Therefore, we have successfully developed the *Agrobacterium*-mediated transient expression co-cultivation process that needs less time, requiring only mixing of cultured plant cells with the recombinant *Agrobacterium* cells, and is suitable for high-throughput analyses and/or rapid, scalable recombinant protein production.

The significant enhancement of rAAT production observed in this study suggests that the lower level of rAAT expression observed in the transgenic CMViva cell cultures is partially due to post-transcriptional gene silencing (PTGS); this is consistent with previous studies [22,25,41,42]. In the transgenic CMViva cell culture, the level of transgene expression is the net balance of long-term transcription and translation limited by PTGS. Although the maximum expression level of rAAT protein was restricted, presumably by RNA silencing, it was significantly enhanced in the presence of viral gene silencing suppressors. Interestingly, the rAAT protein expression was not statistically enhanced by the expression of viral gene silencing suppressors in either the transgenic XVE or the 35S cell suspension cultures, suggesting that viral replicase might play an important role in the production of dsRNA. 

It was observed that the 35S expression system exhibited a very low functional rAAT level, although the total rAAT was higher than that of the XVE and CMV systems in this study (Figure 8). The higher total ATT molecules observed in the 35S expression system might be due to the fact that the AAT gene is being constantly translated for protein expression during the entire growth cycle. It is also presumed that the rAAT is also constantly being converted into a nonfunctional form, either through modifications of the 3D structure (e.g., presentation of the active loop), amino acid modifications, and/or proteolytic cleavage. The overall yields of total and functional rAAT depend on the kinetics of each of these processes. On the other hand, for the CMViva and XVE systems, the production of the AAT transcripts is more synchronized. One hypothesis for this is that the synchronization of transcription afforded by the CMViva system might have more favorable dynamics (e.g., allow maximum accumulation of functional protein). In any case, the lower level of rAAT expression observed in the transgenic CMViva cell cultures is potentially due to post-transcriptional gene silencing (PTGS), consistent with previous studies [22,25,41,42]. Further research may attempt to evaluate the dynamics of the systems at the RNA level.

Previous studies have demonstrated that PTGS within *Agrobacterium*-infiltrated plant leaf tissues clearly suppresses transgene expression level [22,41,42,43,44,45]. In this study, we applied several different viral gene silencing suppressors to individually compare the impact of PTGS on rAAT production in transgenic plant cell cultures (Figure 8). Furthermore, the observed increased rAAT production in transgenic CMViva cell cultures associated with the co-expression of viral gene silencing suppressors of different modes of action has demonstrated that PTGS triggered in the transgenic CMViva cell cultures can be more significantly suppressed by the combination of different viral gene silencing suppressors, resulting in higher transgene expression level (Table 2). These results suggest that individual viral gene silencing suppressors are able to suppress transgene-induced PTGS in induced transgenic CMViva cell culture. However, it seems unlikely that these improvements by a single viral gene silencing suppressor represent a complete elimination of PTGS since the rAAT protein is significantly enhanced in *Agrobacterium*-infiltration co-expressing CP, P19, and P24. Collectively, previous studies indicated that the CP protein of TCV is able to suppress RNA silencing at an early initiation step of PTGS, by interfering with the function of the Dicer-like RNase in plant cells [31]. The P19 protein of TBSV is capable of binding to and sequestering ds siRNA, leading to reduced ss siRNA levels [3,14], and P24 of GLRaV-2 is capable of preventing induction of silencing at the initiation stage, by reducing ds RNA levels [35], suggesting that transgene-induced PTGS can be fully suppressed by combining the modes of action of different viral suppressors.

Specifically, Table 4 compares rAAT production using the transient CMViva system in *N. benthamiana* leaves, with and without co-infiltration, with the P19 gene silencing suppressor [25] and transgenic *N. benthamiana* cell culture in shake flasks in this study (without any viral gene silencing suppressor expression and with P19, CP and P24 co-expression). Recombinant AAT production can be significantly enhanced by transient expression in *N. benthamiana* leaves with the co-expression of the P19 (7.5–19.6 fold increases in functional rAAT protein yield), suggesting that the incorporation of the P19 viral gene silencing suppressor helps to suppress the PTGS pathway, even though the plant tissue is wild type and healthy before applying transient expression. In transgenic CMViva cell cultures, the functional rAAT as a percentage of the total extracellular protein (TSP) increased 5.7 fold with the expression of P19, and 17.2 fold with the co-expression of CP, P19, and P24, representing 26% and 49.9% of the total extracellular rAAT protein, respectively. The specific production level of extracellular functional rAAT on a total soluble protein basis using the transgenic plant cell cultures in shake flask was lower by a factor of about 3.5, compared with transient expression in intact or detached tobacco leaves. The difference in the protein expression systems (i.e., transient expression of AAT and gene silencing suppressor vs. transgenic expression of AAT and transient expression of gene silencing suppressor) and/or host tissue (differentiated leaf vs. plant cell aggregates), as well as the compartment (intracellular vs. extracellular), may be responsible for differences in both the total rAAT expression level, and the percentage of the total rAAT that is functional. However, it is important to recognize that only extracellular rAAT was considered in this work, and there may be a significant amount of functional rAAT that is intracellular and/or cell associated. Furthermore, harvesting of the extracellular rAAT allows for the possibility of continuous bioreactor operation, and it is also possible to harvest part of the plant cell biomass in a semicontinuous operation to recover additional intracellular and/or cell-associated rAAT. An additional consideration is the difference in downstream processing required for the purification of extracellular rAAT from cell culture broth, compared with the purification of intracellular or cell-associated rAAT following homogenization and extraction of plant biomass. It is also possible to incorporate the viral gene silencing suppressors identified in this study in the transgenic host line to improve the rAAT production in transgenic CMViva cell culture. 

In this study, we demonstrated that the *Agrobacterium*-mediated transient expression co-culture process developed by the authors can be a rapid method to evaluate the ability of viral gene silencing suppressors to inhibit transgene-induced PTGS and improve product accumulation in transgenic plant cell cultures. Furthermore, we have shown that the PTGS induced in transgenic CMViva cell culture can be significantly reduced by the transient expression of a single viral gene silencing suppressor, or multiple viral gene silencing suppressors with different modes of action. The *Agrobacterium*-mediated transient co-cultivation process can potentially be used as a platform for (1) the evaluation of new viral suppressors for RNA silencing; (2) the analysis of unidentified genes; and (3) the production of valuable recombinant protein production in plant cell culture. The general method may also be useful for transiently introducing genes into suspension-grown plant cells for a variety of purposes, such as metabolic pathway engineering, modification of glycosylation pathways, and enhancing other properties of viral expression systems. Finally, the approach presented here may be also applicable for enhancing the recombinant proteins production in other transgenic plant cell suspension culture processes [46,47,48].

## 4. Materials and Methods 

### 4.1. Expression Systems Construction and Plant Cell Transformation

The construction of the recombinant vectors, including p35S-spAAT (35S), pXVE-spAAT (XVE), and pCMV-spAAT (CMViva) have been described previously [25]. The three expression systems were stably transformed into *Nicotiana benthamiana* cells using an *Agrobacterium*-mediated transformation approach [49] by utilizing the *Agrobacterium tumefaciens* strain EHA105:pCH32 [50] carrying appropriate binary vectors. 

### 4.2. Transgenic Plant Cell Cultures and Media 

Transgenic *N. benthamiana* cell suspension cultures for each of the three gene expression systems (35S, XVE and CMViva) expressing recombinant human AAT protein were established and analyzed by ELISA and Western blots for rAAT protein [26,27]. The following cell lines, 35S line #0632, XVE line #6011, and CMViva line #8011, producing high levels of extracellular AAT protein, were evaluated for this study [26]. Transgenic *N. benthamiana* cell suspension cultures were sub-cultured every week by transferring 20 mL of the established suspension cells into 200 mL KCMS medium. The KCMS media consist of 30 g/L sucrose, 4.3 g/L MS (Murashige and Skoog) salt mixture, 0.204 g/L KH_2_PO_4_, 0.1 g/L myo-inositol, 10 mg/L thiamine-HCl, 10 mg/L nicotinic acid, 5 mg/L pyridoxine-HCl, 2 mg/L 2,4-D (2,4-dichlorophenoxyacetic acid), and 0.1 mg/L kinetin; the pH 5.8 is adjusted by KOH, in a 1 L flask at 140 rpm and 25 °C.

### 4.3. Biomass Concentration Determination 

Fresh cell weight (FCW) was estimated by filtering a 10 mL cell culture sample onto a Whatman (St. Louis, MO, USA) #1 filter (pre-dried, pre-weighed) connected to a vacuum, washing the cells on the filter with 20 mL of ddH_2_O, and then weighing the cells. Dry cell weight (DCW) was measured after the retained cells were dried on the filter at 60 °C for 2 days [26,27]. 

### 4.4. Agrobacterium Cultures and Viral Gene Silencing Suppressors

Recombinant *Agrobacterium* strains and viral gene silencing suppressors are described in Table 1. All viral gene silencing suppressors were regulated under the CaMV 35S promoter. *A. tumefaciens* cells carrying the corresponding plasmids (Table 1) were grown in 3 mL LB medium containing selection antibiotics (kanamycin at 50 mg/L and rifampicin at 10 mg/L) for 24 h at 28 °C and 250 rpm. For each individual *Agrobacterium* culture, approximately 60 μL (2% inoculation density) was then transferred to sterile tubes containing 3 mL of LB medium supplemented with 1.5 μL of 100 mM acetosyringone (3′,5′-dimethoxy-4′-hydroxyacetophenone) (Aldrich Chemicals, Milwaukee, WI, USA), 60 μL of 1 M 2-(4-morpholino) ethanesulfonic acid (MES) buffer (pH 5.5), and the selection antibiotics grown overnight at 28 °C with shaking at 250 rpm until reaching the cell density (OD_600nm_) of 1.0 to 1.2 (mid-exponential growth phase) measured by a Milton Roy Spectronic 501 spectrophotometer (Milton Roy, New York, NY, USA). *A. tumefaciens* cells were obtained by centrifugation at 2600× *g* for 10 min. The obtained *A. tumefaciens* cells were then re-suspended in sterile de-ionized water, and the bacterial cell density was adjusted to OD_600nm_ of 0.2, as indicated for each experiment in this study. The resuspended recombinant *A. tumefaciens*, carrying the viral gene silencing suppressor solution, was then supplemented with magnesium chloride to reach a final concentration of 10 mM, acetosyringone of 150 μM and MES buffer (pH 5.5) of 20 mM, and incubated at room temperature for three hours in the dark. The corresponding biomass of *Agrobacterium* cells resuspended in sterile water was estimated by the experimental correlation curve of DCW (g/L) vs. OD_600nm_ (g-DCW/L = 0.6051 × OD_600nm_, *R*^2^ = 0.994).

### 4.5. Co-Culture of Agrobacterium with Transgenic Plant Cell Suspensions 

The transient expression co-culture protocol involved growing the transgenic plant cell cultures, 8 days old after inoculation (9% inoculation density) until reaching a biomass concentration of 7-8 g/L (close to the end of exponential phase of cell growth), and the *Agrobacterium* solution (OD_600nm_ of 0.2) together in either 6-well microplate (4 mL working volume) or 250 mL shake flask (50 mL working volume). The resuspended recombinant *A. tumefaciens* solution (OD_600nm_ of 0.2) was added to plant cell cultures at a variety of biomass ratios of *Agrobacterium* cells to plant cells (μg-DCW-*Agrobacterium* cells)/(g-DCW-plant cells), as indicated. The plant cells and *Agrobacterium* were co-cultured at 20 °C with shaking at 40 rpm for the first 24 h; speed was then increased to 140 rpm for the following cultivation. For inducible plant cell cultures (XVE and CMViva), induction was initiated at 1 or 2 days after starting co-culture process. 

### 4.6. Induction Treatment

The 17 β-estradiol (Sigma Inc., St. Louis, MO, USA) was prepared in DMSO (dimethyl sulfoxide) as the chemical inducer solution used in this study. To initiate the induction phase, the inducer solution was added to transgenic XVE and CMViva plant cell cultures at a specific time post-inoculation with a final inducer concentration of 10 μM. No inducer solution was applied to the transgenic 35S plant cell culture transformed with the 35S constitutive promoter [26,27]. 

### 4.7. Protein Quantitative and Qualitative Analysis

The protein analysis methods used in this study, including total AAT ELISA, functional AAT ELISA and total soluble protein (TSP), were executed as described previously [25]. In short, the method of total AAT ELISA was developed using the sandwich ELISA principle, in which rabbit anti-human α1-antitrypsin polyclonal IgG fraction (Dako A/S, Glostrup, Denmark) was used as the capture antibody, horseradish peroxidase (HRP)-conjugated polyclonal goat anti-human α1-antitrypsin IgG (US Biological, Swampscott, MA, USA) was used as secondary antibody, and SureBlue peroxidase (KPL, Gaithersburg, MD, USA) was used as substrate solution. The reaction was terminated by the addition of 1 N HCl solution. The absorbance at 450 nm was then measured using a SpectraMax 340pc microplate reader (Molecular Devices, Sunnyvale, CA, USA). To generate ELISA standard curve, human α1-antitrypsin (Calbiochem, La Jolla, CA, USA) was used in this study. The functional AAT ELISA has been validated using a “band shift assay”, as shown in previous study [25]. The method of functional AAT ELISA was developed using the same ELISA protocol as the total AAT assay, except for the two following modifications. (1) Human AAT standard and samples were mixed with porcine pancreatic elastase (PPE) as a substrate for AAT (Calbiochem, Temecula, CA, USA) and then incubated at 37 °C for 20 min to allow the AAT-PPE complex formation (a reaction product of AAT binding irreversibly to PPE). (2) A polyclonal rabbit anti-elastase IgG conjugated to HRP (US Biological, Swampscott, MA, USA) was used as secondary antibody to allow specific detection of the AAT-PPE complex. Biologically functional AAT is defined as AAT capable of irreversible binding to PPE [26,27]. 

### 4.8. Analysis of the Impact of Viral Gene Silencing on PTGS 

The effect of viral gene silencing suppressors on reducing transgene-induced PTGS and on improving the rAAT production within *Agrobacterium*-infiltrated transgenic plant cell cultures is estimated by an increase in rAAT product yield and functionality associated with transient expression of the viral gene silencing suppressor of interest, and is compared to samples which are not co-cultured with recombinant *Agrobacterium* cells or co-cultured with recombinant *Agrobacterium* cells containing 35S CaMV promoter driving GUS expression. The experiments for Figure 1, Figure 2, Figure 3, Figure 4, Figure 5, Figure 6 and Figure 7 were carried out with one replicate, and the results shown were the average of two sample assays of one replicate experiment. The experiments for Figure 8 and Figure 10 were carried out independently in duplicates, and the results shown were the average of three replicate sample assays of duplicate experiments. A student’s *t* test was used for statistical hypothesis testing between test and control groups for the data presented in Figure 8 and Figure 10.

### 4.9. Fractional Factorial Design (FFD)

Fractional factorial design, which consists of a chosen fraction of the experimental runs of a full factorial design, was applied to screen critical variables (viral gene silencing suppressor) in this study. The two-level fractional factorial design matrix was created by Design-Expert version 7.0 software (Stat-Ease, Inc., Minneapolis, MN, USA) and the experimental runs were carried out according to the design matrix. Statistical analyses were made to identify variables that had a significant positive effect or negative effect on rAAT production yield. The variables with confidence levels above 95% were considered to have significantly influenced rAAT production. 

### 4.10. Response Surface Methodology (RSM)

RSM using full-factorial central composite design (CCD) [51] was used to optimize the values of identified variables (biomass ratio of recombinant *Agrobacterium* carrying viral gene silencing suppressor to transgenic plant cells) for enhancing rAAT production. The RSM-CCD design matrix was created by Design-Expert software, and the experimental runs were carried out according to the design matrix. Statistical analyses were made to identify the optimal levels of variables for maximizing rAAT production yield. The variables with confidence levels above 95% were considered to have significantly influenced rAAT production.

## 5. Conclusions

In this study, we demonstrated that the *Agrobacterium*-mediated transient expression co-culture process can serve as a rapid method to evaluate the effect of viral gene silencing suppressors for inhibiting transgene-induced PTGS, and to improve product accumulation in transgenic plant cell cultures. Furthermore, we have shown that the PTGS induced in transgenic CMViva cell culture can be significantly reduced by the transient expression of a single viral gene silencing suppressor, or by multiple viral gene silencing suppressors with different modes of action. The *Agrobacterium*-mediated transient expression co-cultivation process can be used to identify new viral suppressors of RNA silencing, for detailed analysis of unidentified genes, and for the production of valuable recombinant proteins production in plant cell cultures. The general method may also be useful for transiently introducing genes into suspension-grown plant cells for a variety of purposes, such as metabolic pathway engineering, modification of glycosylation pathways, and enhancing other properties of viral expression systems.

## Figures and Tables

**Table 1:** Viral gene silencing suppressors of RNA silencing investigated in this work.

*Agrobacterium* Strain	Gene Silencing Suppressor	Virus	Potential Modes of Action
EHA105	2b	*Cucumber mosaic virus* (CMV)	Required for host-specific movement of PTGS signals [29]; Interacts with components of the RISC machinery to reduce ARGONAUTE (AGO) cleavage activity [30].
GV2260	coat protein (CP) (also referred to as p38)	*Turnip crinkle virus* (TCV)	TCV CP functions to suppress RNA silencing at an early initiation step of PTGS by interfering with the function of the Dicer-like RNase in plants [31].
GV2260	HC-Pro	*Tobacco etch virus* (TEV)	Functions by binding to double-stranded siRNA (ds siRNA) and inhibits their unwinding to single-stranded siRNA (ss siRNA) [13].
EHA105	P1	*Rice yellow mottle virus* (RYMV)	P1 of RYMV is required for systemic virus spread and movement [32].
GV2260	P10	*Grapevine virus A* (GVA)	P10 of GVA reduces the levels of ss siRNAs by sequestering ds siRNAs [33].
EHA105	P19	*Tomato bushy stunt virus* (TBSV)	P19 of TBSV functions by binding to and sequestering ds siRNA, reducing the ss siRNA level [3,10,14].
GV2260	P21	*Beet yellow virus* (BYV)	P21 silencing suppression mechanism is similar to P19 for inhibiting silencing pathways by binding ds siRNA [34].
GV2260	P24	*Grapevine leafroll associated virus-2* (GLRaV-2)	P24 of GLRaV-2 is capable of preventing induction of silencing by double-stranded inverted repeat, reducing the ds RNA levels [35].
EHA105	P25	*Potato virus X* (PVX)	P25 of PVX is responsible for cell-to-cell movement of PTGS signals and blocks systemic silencing [36].

**Table 2:** Two level fractional factorial design variables (viral gene silencing suppressors) with recombinant human AAT production as response, and its ANOVA result (analysis of variance and regression analysis).

Run No.	Viral Gene Silencing Suppressors (μg-*Agrobacterium*/g-Plant Cell Biomass)					Extracellular Total rAAT (μg/L)	Extracellular Functional rAAT (μg/L)	Functional/Total rAAT (%)
	CP	P19	P21	P24	P25			
1	0	0	0	0	10	327.7	100.1	30.54
2	10	0	0	0	0	227.8	92.9	40.77
3	0	10	0	0	0	278.3	95.2	34.22
4	10	10	0	0	10	247.4	104.4	42.22
5	0	0	10	0	0	255.3	120.8	47.31
6	10	0	10	0	10	302.9	112.0	36.98
7	0	10	10	0	10	307.7	85.7	27.86
8	10	10	10	0	0	326.0	134.1	41.14
9	0	0	0	10	0	265.0	103.5	39.05
10	10	0	0	10	10	245.4	114.5	46.65
11	0	10	0	10	10	311.1	108.1	34.76
12	10	10	0	10	0	556.2	250.3	45.00
13	0	0	10	10	10	539.4	243.1	45.07
14	10	0	10	10	0	347.4	194.4	55.95
15	0	10	10	10	0	339.9	110.5	32.51
16	10	10	10	10	10	301.8	120.1	39.79
Control	0	0	0	0	0	121.8	13.1	10.79

ANOVA for extracellular total rAAT model: *R*^2^ = 0.999; CV = 0.97%; Std. Dev. = 3.13; *F* value = 974.44; *p* value = 0.0251 (≤0.05); ANOVA for extracellular functional rAAT model: *R*^2^ = 0.996; CV = 6.1%; Std. Dev. = 7.97; *F* value = 48.05; *p* value = 0.0206 (≤0.05).

**Table 3:** Central composite design matrix of three variables (CP, P19 and P24) with experimental and predicted values of recombinant human AAT production.

Run No.	Viral Gene Silencing Suppressors (μg-*Agrobacterium*/g-Plant Cell)			Extracellular Total rAAT (μg/L)		Extracellular Functional rAAT (μg/L)	
	CP	P19	P24	Experimental Value	Predicted Value	Experimental Value	Predicted Value
1	8	8	8	332.8	363.3	176.3	157.6
2	25	8	8	359.1	320.6	167.5	184.8
3	8	25	8	361.4	306.1	157.8	164.6
4	25	25	8	260.0	252.1	192.1	195.1
5	8	8	25	250.2	211.1	159.4	172.2
6	25	8	25	325.4	333.8	184.9	193.7
7	8	25	25	351.4	343.0	214.0	212.4
8	25	25	25	618.4	454.5	301.6	237.3
9	0	17	17	321.9	302.2	193.7	164.9
10	34	17	17	304.3	371.0	203.9	217.0
11	17	0	17	288.6	252.1	184.1	144.8
12	17	34	17	232.1	315.6	171.9	195.4
13	17	17	0	231.5	211.2	176.1	142.8
14	17	17	34	194.0	261.3	181.9	199.5
15	17	17	17	323.7	435.5	168.2	226.6
Control	0	0	0	216.5	238.1	15.5	30.2

**Table 4:** Comparison of rAAT production in *N. benthamiana* using the CMViva system following induction in *N. benthamiana* leaves through *Agrobacterium*-mediated transient expression and transgenic *N. benthamiana* cell cultures. TSP = total soluble protein.

CMViva System	Functional rAAT/TSP (%)	Total rAAT/TSP (%)	Functional AAT/Total AAT (%)
Transient expression in whole intact plant leaves, without p19, topical infiltration and induction [25]	0.16	0.57	28.1
Transient expression in whole intact plant leaves, with p19, topical infiltration and induction [25]	1.2	1.7	70.5
Transient expression in detached plant leaves, without p19, vacuum infiltration and induction [43]	0.061	0.364	16.7
Transient expression in detached plant leaves, with p19, vacuum infiltration and induction [43]	1.196	4.068	29.4
Transient expression in detached plant leaves, with p19, optimized vacuum infiltration and induction [44]	2.6	4.1	63.4
Transgenic plant cell cultures in shake flask, without p19 transient expression (extracellular rAAT yield), this work	0.0195	0.163	11.9
Transgenic plant cell cultures in shake flask, with p19 transient expression (extracellular rAAT yield), this work	0.110	0.422	26.1
Transgenic plant cell cultures in shake flask, with transient co-expression of CP, P19 and P24 (extracellular rAAT yield), this work	0.336	0.673	49.9
